# Potential correlates of burnout among general practitioners and residents in Hungary: the significant role of gender, age, dependant care and experience

**DOI:** 10.1186/s12875-018-0886-3

**Published:** 2018-12-12

**Authors:** Szilvia Adam, Andras Mohos, Laszlo Kalabay, Peter Torzsa

**Affiliations:** 1 Institute of Behavioural Sciences, Semmelweis University, Budapest, Hungary; 2 Department of Family Medicine, Faculty of Medicine ,University of Szeged, Szeged, Hungary; 3 Department of Family Medicine, Medical Faculty, Semmelweis University, Budapest, Hungary

**Keywords:** Burnout, General practitioners, Residents, Gender, Correlates, Predictors

## Abstract

**Background:**

Burnout is increasingly prevalent among general practitioners (GPs) in Hungary, which may lead to functional impairment and, subsequently, to poor quality of patient care. However, little is known about potential predictors of burnout among GPs. The aim of this study was to explore psychosocial correlates of burnout among GPs and residents in Hungary.

**Methods:**

We collected socio-demographic and work-related data with self-administered questionnaires in a cross-sectional study among GPs (*N* = 196) and residents (*N* = 154). We assessed burnout with the Maslach Burnout Inventory Human Services Survey (MBI-HSS) and calculated the mean level of burnout and the proportion of physicians suffering from low, intermediate and high degree of burnout. To identify potential socio-demographic and work-related correlates of burnout among physicians, we determined Spearman’s and Mann-Whitney U correlation coefficients and conducted stepwise linear regression analyses. We deployed Mann-Whitney U test to explore gender disparity in the level of burnout between female and male physicians and between general practitioners and residents.

**Results:**

The prevalence of moderate to high level emotional exhaustion, depersonalisation, and impaired personal accomplishment was 34.7, 33.5 and 67.8% as well as 41.0, 43.1, and 71.1% among GPs and residents, respectively. Residents reported significantly lower level of personal accomplishment vs GPs. We identified a significantly higher level of depersonalization among male physicians compared to female physicians. Age correlated negatively with emotional exhaustion and depersonalization and positively with personal accomplishment among GPs. Dependant care was positively associated with burnout among female GPs. Female residents were more likely to report depersonalization. High workload was positively correlated with depersonalization among female GPs. Younger age emerged as the strongest predictor of emotional exhaustion. Male gender and fewer years of experience predicted depersonalization best, and male gender showed a significant predictive relationship with low personal accomplishment.

**Conclusion:**

We identified specific socio-demographic and work-related correlates of burnout, which may guide the development of specific and effective organizational decisions to attenuate occupational stress and subsequent burnout as well as functional impairment among GPs, and thus, may improve the quality of patient care.

## Background

The well-being, defined as good mental and physical health, of general practitioners (GPs) is essential to maintaining an effective health care system as they are in the front line to guard and promote health, advocate and administer preventive care, diagnose new illnesses, and manage chronic ones.

However, research on the mental health of GPs in various countries has revealed high prevalence of distress, burnout, suicidal ideation, alcohol and substance misuse, anxiety, and depression [1–3], which have been associated with functional impairment and, subsequently, with poor quality of patient care [4, 5]. Furthermore, several studies demonstrated that the prevalence of burnout among residents ranges between 28.0 and 45.0% [6–8].

Burnout among GPs is of particular concern as there are many characteristics of contemporary general practice that increase the risk of burnout such as the high number of patient-doctor contacts, increased administrative burden, work stress due to high demands from patients and colleagues, reduced support from family and colleagues, as well as limited autonomy in decision-making [9–11]. Burnout is defined as a syndrome of emotional exhaustion, depersonalisation, and reduced personal accomplishment originally described by Maslach, Jackson, and Leiter [12], which develops as a response to chronic emotional or interpersonal distress.

Research in various countries has identified individual (e.g., behavioural, demographic, and personality-related), organisational, as well as societal determinants of burnout among physicians. For example, physicians often go to work even when they are ill [13] or make their own diagnoses and prescribe their own treatment [14]. Physicians rarely seek help for their ailments from other medical professionals and when they do so it is often after a long delay when their health condition has worsened [15]. Further individual risk factors are two personality types, neuroticism and perfectionism, which may play a significant role in the development of burnout through the deployment of maladaptive coping mechanisms for work stress [16]. Amongst demographic factors, age, dependant care demands such as large numbers of children and elderly people in the family requiring care, as well as gender have been identified as important determinants of burnout [17]. Whilst in the US burnout tends to occur most frequently among young employees [12], in Europe burnout seems to be more prevalent among older employees [18], which could be explained by limited workforce mobility in Europe. However, recent research in Europe has identified a trend of higher burnout levels among younger physicians compared to older ones [19].

The role of gender in the development of burnout has been extensively studied. While the results are inconclusive, in general, women score higher on emotional exhaustion, and men score higher on depersonalisation and personal accomplishment. This trend has also been described among physicians [20].

Amongst workplace characteristics, high job demands, role conflict, low job resources, and lack of workplace support have been identified as key risk factors of burnout among GPs and physicians [21–23]. Furthermore, our research among GPs in Hungary and studies in other European countries have shown that psychosocial stress affects the mental well-being of GPs and contributes to increased prevalence of burnout, anxiety, and depression [24–31].

In the Hungarian context, research consequently showed low personal accomplishment among nearly 100.0% of GPs and residents surveyed. Our previous study also revealed high level of depersonalisation and emotional exhaustion among 61.0 and 35.0% of the GPs, respectively. The proportion of Hungarian physicians with high levels of depersonalisation and low personal accomplishment is significantly higher than those obtained among Italian, Swiss, Spanish, and Canadian physicians [32].

Our research group has shown that in the Hungarian context, physician burnout is closely associated with workplace stress, and stress-related somatic diseases such as gastrointestinal and cardiovascular disorders [33]. Furthermore, in-depth interviews conducted among Hungarian physicians revealed that the causes of increased workplace stress were associated with reduced control over work (control over the amount of work and the number of patients), flaws of the organisational structure (poor communication, lack of cooperation, lack of support from more experienced physicians) and with stress resulting from unique work characteristics (e.g., high responsibility, failed attempts of treatment, long working hours). We also identified a further antecedent to workplace stress, namely work-family conflict, and found it as a strong correlate of burnout among female and male physicians in Hungary [34, 35]. Our findings showed that the relative importance of the work domain in the development of work-family conflict within the Hungarian medical profession is more significant than that of the family domain. Because in Hungary men and women tend to adhere to traditional gender roles, female physicians need to cope with the supremacy of their work domain as a professional and with the significance of the family domain as a woman. These findings suggest that work and family-related factors are critically important in the pathogenesis of stress and stress-related morbidities including burnout and other somatic diseases among physicians in Hungary [36, 37]. Whilst these relationships have been extensively studied among physicians, in general, little attention has been paid to identifying potential risk factors of burnout among GPs in particular. Given the pivotal role of GPs and residents in improving or maintaining public health, there is a strong rationale to explore psychosocial determinants and/or correlates of burnout among GPs and residents in Hungary. Based on transactional and interactional models of burnout as a theoretical framework [38, 39], the hypotheses of this research are as follows:

1. The prevalence of burnout among Hungarian GPs and residents is high.
2. Female GPs and residents experience significantly higher level of burnout compared to their male counterparts.
3. Older age is associated with burnout among GPs and residents.
4. High number of children (a surrogate of dependant care) is associated with burnout among female physicians.
5. High number of registered patients in the practice (a surrogate of workload) is associated with burnout among GPs and residents.

## Methods

### Study design, setting, and participants

We used a cross-sectional design and self-administered questionnaires, which we distributed to 346 general practitioners and 234 residents, who attended a Continued Medical Education (CME) course. Of the 580 participants, 350 general practitioners returned the appropriately completed questionnaire (196 general practitioners (56.0%) and 154 residents (44.0%)) and thus were selected for the study.

## Measures

We used the Maslach Burnout Inventory Human Services Survey (MBI-HSS) [12]. This 22-item instrument has three subscales and measures (1) emotional exhaustion with 9 items, (2) depersonalization (alienation) from work, patients, and colleagues with 5 items, and (3) personal accomplishment which indicates job-related competency and subsequent self-esteem with 8 items. Responses are marked on a seven-point Likert scale (0 meaning ‘never’ and 6 meaning ‘every day’) and then summed. A high degree of burnout among general practitioners was reflected by sum scores of ≥27 on the emotional exhaustion and ≥ 10 on the depersonalization subscales, and a score of ≤33 on the personal accomplishment subscale, which represent the cut-off scores at the upper tercile of the sample [12]. The reliability coefficients (Cronbach’s alpha) of the emotional exhaustion, depersonalization, and personal accomplishment scales for our total sample were 0.9, 0.8, and 0.8, respectively. Similar reliability coefficients on all subscales have been obtained for female and male physicians, and residents as well as GPs.

We collected sociodemographic data such as gender, age, number of children (as a surrogate of dependant care), and marital status. We also assessed workplace factors including type of practice (adult, paediatric, or mixed), number of patients in the practice (as a surrogate of workload) defined by the number of registered patients (social security cards) in the practice, years spent in the practice (as a surrogate of experience), and regular on-call duties over the week-end (at least once a month) as well as nightshift (defined as work hours between 7 PM and 7 AM the following day at least once a week).

### Data analyses

We calculated mean scores and standard deviation (SD) for each of the three scales of burnout for the whole sample, for GPs and residents, as well as for female and male GPs. To explore differences in the mean scores of each burnout dimension between female and male physicians as well as general practitioners and residents, we deployed the Mann-Whitney U test.

We classified physicians into three groups based on the perceived level of burnout (high, intermediate, low) based on cut-off values reported by Maslach et al. [12]. To identify correlates of burnout among physicians, we assessed correlations among socio-demographic and work-related characteristics (i.e., age, marital status, number of children, job title (GP or resident), number of patients in the practice, average of years spent in the practice, specialty focus (e.g., paediatrics, adults, mixed), and on-call duties over the week-end or nightshifts) and the three dimensions of burnout experienced by male and female physicians as well as the total sample by determining Spearman’s (r_S_) and Mann-Whitney U correlation coefficients for continuous and dichotomous dependent variables, respectively.

In order to explore the relationship between burnout and socio-demographic as well as work-related factors further, we conducted stepwise linear regression analyses and assessed the strength and direction of relationships between the continuous dependent variables (emotional exhaustion, depersonalization, personal accomplishment) and explanatory variables (age, gender, marital status, number of children, number of patients in the practice, average years spent in the practice, specialty, on-call over week-ends, nightshifts, and job title (GP vs resident)) by determining regression coefficients (adjusted β), 95% confidence intervals (95% CI), and t-test statistics. We used F statistics to confirm the significance of models at each step. We controlled for gender and type of employment (inpatient/outpatient services, general practice, and other establishments) in the analyses. In addition, we calculated the proportion of the variance in the dependent variable explained by the explanatory variables (adjusted R^2^). One model for emotional exhaustion, two for depersonalization and one for personal accomplishment have been examined. The models were significant for each step as determined by F statistics (data not shown). A *p* value of < 0.05 was considered as statistically significant. The statistical software used for all analyses was SPSS, version 20.0 (IBM-SPSS Inc., Armonk, NY, USA).

## Results

### Participant characteristics

The overall response rate was 60.3%. Table 1 shows the demographic profile of the sample. The average age (SD) was 45.6 years (15.0) (age ranging from 25 to 79 years). Most of the physicians were partnered (78.2%) and had children (67.1%). The average number of patients in the practice was similar among male and female physicians. The average (SD) of years spent in the practice was 12.0 (12.7) years. Only around one fifth and one third of the female and male physicians, respectively, took on-call duty over the week-end and night-shift (Table 1). GPs and residents in our sample represent 3.0% of the total number of GPs (6541) and 66.0% of residents (234) in Hungary, respectively. In the normative population, the proportion of female GPs is 68.0% and most GPs are in the 50–59-year-old cohort. The distribution of GPs among adult, paediatric, and mixed practices is 54.0, 23.0, and 23.0% in Hungary. Our study sample may be representative of the normative population in terms of gender, age distribution, residents, and GPs focusing on adult patients.

**Table 1:** Sociodemographic characteristics of the sample

Variable	Total sample^a^ N (%)	Female^a^ N (%)	Male^a^ N (%)
Total sample	350 (100.0)	209 (59.7)	141 (40.3)
General practitioners	196 (56.0)	108 (51.7)	88 (32.4)
Residents	154 (44.0)	101 (48.3)	53 (37.6)
Age group (years)			
< 30	88 (25.3)	58 (28.0)	30 (21.3)
30–39	53 (15.2)	34 (16.4)	19 (13.4)
40–49	50 (14.4)	30 (14.5)	20 (14.2)
50–59	80 (23.0)	42 (20.3)	38 (27.0)
≥ 60	77 (22.1)	43 (20.8)	34 (24.1)
Partner			
Yes	248 (78.2)	134 (73.2)	114 (85.1)
No	69 (21.8)	49 (26.8)	20 (14.9)
Number of children			
None	114 (32.9)	71 (34.0)	43 (31.2)
Yes	233 (67.1)	138 (66.0)	95 (68.8)
1	56 (24.0)	36 (26.1)	20 (21.1)
2	105 (45.1)	69 (50.0)	36 (37.9)
≥ 3	72 (30.9)	33 (23.9)	39 (41.0)
Number of registered patients in GP practice (Mean (SD))	1813.9 (534.2)	1738.9 (546.6)	1900.9 (508.7)
Type of GP practice			
Adult	291 (72.9)	155 (74.2)	113 (80.1)
Paediatric	32 (8.0)	28 (13.4)	4 (2.8)
Mixed	43 (10.8)	19 (9.1)	139 (98.6)
Missing data	33 (8.3)	7 (3.3)	2 (1.4)
Average years in GP practice (Mean (SD))	12.0 (12.7)	11.9 (12.5)	14.1 (13.3)
On-call over week-end, nightshift			
No	143 (73.0)	86 (81.1)	57 (64.8)
Yes	51 (26.0)	20 (18.9)	31 (35.2)
Missing data	2 (1.0)		

^a^General practitioners and residents combined

### High prevalence of burnout among general practitioners and residents

In line with our first hypothesis, a sizeable proportion of GPs and resident reported high level of burnout. Among GPs, 19.9, 18.0, and 45.9% reported high level of emotional exhaustion, depersonalization, and impaired personal accomplishment, respectively. The prevalence of high level of emotional exhaustion, depersonalization, and impaired personal accomplishment among residents was similar (18.5, 25.5, and 39.5%, respectively). Moderate to high levels of emotional exhaustion and depersonalization were reported by 34.7 and 33.5% as well as by 41.0 and 43.1% of GPs and residents, respectively. Moderate to high level impaired personal accomplishment was also prevalent among GPs and residents (67.8 and 71.1%, respectively) (Table 2).

**Table 2:** Prevalence of burnout among general practitioners (*N* = 196) and residents (*N* = 154)

Variable	High level		Moderate level		Low level		Mann-Whitney U-test
	GPs	Residents	GPs	Residents	GPs	Residents	
Burnout N (%)							
Emotional exhaustion	39 (19.9)	28 (18.5)	29 (14.8)	34 (22.5)	128 (65.3)	89 (58.9)	NS^a^
Depersonalization	35 (18.0)	39 (25.5)	30 (15.5)	27 (17.6)	129 (65.5)	87 (56.9)	NS^a^
Impaired personal accomplishment	90 (45.9)	60 (39.5)	43 (21.9)	48 (31.6)	63 (32.1)	44 (28.9)	U = 12,247.0**

^a^NS: Not significant

** *p* < 0.01

Whilst we observed no significant differences in the level of emotional exhaustion and depersonalization between GPs and residents, residents reported significantly lower level of personal accomplishment compared to GPs (Mann-Whitney U = 12,247.0; *p* < 0.01) (Table 2).

### Gender disparity in the level of depersonalization between female and male physicians

In contrary to our second hypothesis, our results demonstrated a significantly higher level of depersonalization among male physicians compared to female physicians (Mann-Whitney U = 12,221.0; *p* < 0.01). We found no significant gender differences in the levels of emotional exhaustion and personal accomplishment (Table 3).

**Table 3:** Gender differences in the level of burnout among female (*N* = 209) and male (*N* = 141) physicians

Burnout	Female physicians	Male physicians	Mann-Whitney U-test
Emotional exhaustion Mean Rank	170.4	179.4	NS^a^
Depersonalization Mean Rank	163.0	190.2	U = 12,221.0**
Personal accomplishment Mean Rank	177.7	169.7	NS^a^

^a^*NS* Not significant

**p < 0.01

### Socio-demographic and work-related correlates of burnout among GPs and residents

As demonstrated in Table 4, we identified that age negatively correlated with both emotional exhaustion and depersonalization among female physicians (r_S_ = − 0.193, *p* < 0.01; r_S_ = − 0.220, *p* < 0.001), male physicians (r_S_ = − 0.206, *p* < 0.01; r_S_ = − 0.191, *p* < 0.05), and in the total sample (r_S_ = − 0.186, *p* < 0.01; r_S_ = − 0.198, *p* < 0.001), respectively. Age correlated positively with personal accomplishment among female physicians (r_S_ = 0.358, *p* < 0.001) and the total sample (r_S_ = 0.234, *p* < 0.001) but not among male physicians. These results are contrary to our third hypothesis, which stipulated that age was a potential positive correlate of burnout among GPs and residents.

**Table 4:** Potential socio-demographic and work-related correlates of burnout among female (*N* = 209) and male (*N* = 141) physicians

	Emotional exhaustion			Depersonalization			Personal accomplishment		
Variables	Female physicians	Male physicians	Total sample	Female physicians	Male physicians	Total sample	Female physicians	Male physicians	Total sample
Age	r_S_ = −0.193^**^	r_S_ = − 0.206^**^	r_S_ = − 0.186^**^	r_S_ = − 0.220^***^	r_S_ = − 0.191^*^	r_S_ = − 0.198 ^***^	r_S_ = 0.358^***^	NS^a^	r_S_ = 0.234^***^
Marital status	NS^a^	NS^a^	NS^a^	NS^a^	NS^a^	NS^a^	NS^a^	NS^a^	NS^a^
Number of children	r_S_ = 0.159^*^	NS^a^	r_S_ = 0.119^*^	r_S_ = 0.141^*^	NS^a^	r_S_ = 0.117^*^	r_S_ = − 0.178^**^	NS^a^	NS^a^
Job title (GP vs resident)	NS^a^	NS^a^	NS^a^	U = 4461.5^*^	NS^a^	NS^a^	U = 3823.5^***^	NS^a^	U = 12,247.0^**^
Number of patients in the practice	NS^a^	NS^a^	NS^a^	r_S_ = 0.216^*^	NS^a^	NS^a^	NS^a^	NS^a^	NS^a^
Average of years spent in the practice	NS^a^	NS^a^	NS^a^	r_S_ = − 0.176^**^	NS^a^	r_S_ = − 0.133^**^	r_S_ = 0.264^***^	NS^a^	r_S_ = 0.168^**^
Specialty focus of GPs and residents	NS^a^	NS^a^	NS^a^	NS^a^	NS^a^	NS^a^	NS^a^	NS^a^	NS^a^
On-call over week-end, nightshifts	NS^a^	NS^a^	NS^a^	NS^a^	NS^a^	NS^a^	NS^a^	NS^a^	NS^a^

^a^*NS* Not significant

* *p* < 0.05; ** *p* < 0.01, *** *p* < 0.001

In line with our fourth hypothesis, we found that the number of children (a surrogate of dependant care) was a significant positive correlate of emotional exhaustion (r_S_ = 0.159; *p* < 0.05) and depersonalization (r_S_ = 0.141, *p* < 0.05), and a negative correlate of personal accomplishment (r_S_ = − 0.178, *p* < 0.01) among female physicians. In addition, the number of children had a positive association with emotional exhaustion (r_S_ = 0.119, *p* < 0.05) and depersonalization (r_S_ = 0.117, *p* > 0.05) in the total sample (Table 4).

Our results showed that among female physicians, being a resident was significantly correlated with depersonalisation (mean ranks were 112.9 vs 95.7 among residents vs GPs, respectively, Mann-Whitney U = 4461.5, *p* < 0.05) and being a general practitioner was significantly correlated with personal accomplishment (mean ranks were 118.1 vs 88.6 among GPs vs residents, respectively, Mann-Whitney U = 3823.5, *p* < 0.001). In addition, being a GP was a significant positive correlate of personal accomplishment in the total population (mean ranks were 188.0 vs 157.0 among GPs vs residents, respectively, Mann-Whitney U = 12,247.0, *p* < 0.01) (Table 4).

We found that the number of registered patients in the practice (a surrogate of workload) was significantly associated with depersonalization (r_S_ = 0.216, *p* < 0.05) among female physicians. These results confirm, at least in part, our final hypothesis, as we could demonstrate associations between the number of patients in the practice and burnout, albeit only for one burnout dimension (depersonalization) and among female physicians only. Furthermore, the average of years spent in the practice (a surrogate of experience) was a significant inverse correlate of depersonalization among female physicians (r_S_ = − 0.176, *p* < 0.01) and the total sample (r_S_ = − 0.133, *p* < 0.01) and a positive correlate of personal accomplishment among female physicians (r_S_ = 0.264, *p* < 0.001) and in the total sample (r_S_ = 0.168, *p* < 0.01). We did not find significant correlations between burnout and a few other sociodemographic and work-related variables such as marital status, specialty focus (i.e., paediatric or adult patients or a combination thereof), and on-call duties over the week-end or nightshifts (Table 4).

The results of our regression analyses showed that of the variables examined (age, gender, marital status, number of children, number of patients in the practice, average years spent in the practice, specialty, on-call over week-ends, nightshifts, and job title (GP vs resident)), younger age (β = − 0.153, 95% CI -0.343 – -0.01) predicted emotional exhaustion best. Male gender (β = − 0.211, 95% CI -3.974 – -0.788) and fewer years spent in practices (β = − 0.156, 95% CI -0.156 – -0.008) emerged as best predictors of depersonalization. Low personal accomplishment was best predicted by male gender (β = 0.177, 95% CI 0.541–5.139). These predictors explained 2.3, 2.4, and 3.1% of the variance in emotional exhaustion, depersonalization and low personal accomplishment, respectively (Table 5).

**Table 5:** Potential predictors associated with burnout (emotional exhaustion, depersonalization and reduced personal accomplishment) among general practitioners and residents: stepwise linear regression analyses

Dependent variables	Independent variables	Standardized β	95% Confidence intervals	t	Adjusted R^2^
Emotional exhaustion	Age (younger)	−0.153	− 0.343 – − 0.010	−2.096*	0.023
Depersonalization	Gender (male)	−0.211	−3.974 – − 0.788	−2.949**	0.024
	Average years spent in practice (shorter)	−0.156	−0.156 – − 0.008	−2.174*	
Personal accomplishment	Gender (female)	0.177	0.541–5.139	2.438*	0.031

* *p* < 0.05, ** *p* < 0.01

## Discussion

In this study, we found high prevalence of burnout and significant gender disparity among Hungarian GPs and residents. Residents reported significantly higher level of burnout (low level of personal accomplishment) vs GPs. In addition, we determined various psychosocial correlates of burnout and identified younger age, male gender, and fewer years of experience as significant predictors of specific burnout dimensions among the physicians.

The primary goal of our study was to assess the prevalence of burnout in a sample of GPs and residents in Hungary. In line with our first hypothesis, our results suggest that burnout is relatively prevalent in this population, with 18.0–25.5% reporting a high level of emotional exhaustion and depersonalization, and 39.5–45.9% of the sample reporting a high level of impaired personal accomplishment. The comparison of our findings with the results from studies of burnout among Italian [40], UK [41], European [42], Irish [43], Canadian [44], and Slovenian [45] GPs revealed that Hungarian GPs reported comparatively lower levels of emotional exhaustion and depersonalization, but significantly higher level of impaired personal accomplishment. Our findings about the high prevalence of impaired personal accomplishment among GPs and residents are in line with the results of our research among physicians in Hungary. We posit that low level of personal accomplishment could develop independently and in parallel with exhaustion [46]. This may be observed in certain organisational environments characterised by role conflict or work overload that on the one hand intensify emotional exhaustion and on the other hand simultaneously reduce personal accomplishment through disabling participative decision making and social support, which serve as significant facilitators of personal accomplishment [35]. Our results support the growing body of evidence which has found increasing prevalence of burnout among GPs all over the world.

Another objective of our study was to identify correlates (or potential predictors) of burnout among GPs and residents in Hungary focusing on socio-demographic and work-related factors. Based on a multitude of evidence, it is generally suggested that female employees may be more susceptible to experiencing burnout. In particular, data suggest that women may be more prone to experiencing a greater level of emotional exhaustion than men and that men more frequently report depersonalization than women. Our results confirm these findings. Whilst we found a significantly higher mean level of depersonalization among male GPs, we did not see a difference in emotional exhaustion. In addition, our results suggest that male GPs and residents might be more likely to report depersonalization and reduced personal accomplishment than women. However, data in the literature appear to be inconclusive regarding the strength and direction of gender differences in burnout and only a handful of authors have explored this relationship directly. In a recent meta-analysis of the relationship between gender and burnout, results demonstrated that women were slightly more emotionally exhausted than men, while men are somewhat more depersonalized than women [47]. In line with our hypothesis, our results confirm, at least in part, these findings. Our findings underpin the significance of gender research regarding burnout because they would contribute to making adequate and accurate organizational decisions to reduce occupational stress and consequent burnout.

Of all the demographic variables, age has been extensively studied and most consistently related to burnout [48]. Several studies have suggested that younger employees experience higher level of burnout than older ones. Age is associated with work experience, and it has been postulated that younger employees have less work experience, which may lead to higher level of occupational stress and subsequent burnout. Indeed, research has shown a decrease in the burnout scores of all three dimensions as the subjects’ age increased [49]. However, others have found no significant differences in burnout among different age ranges [50]. In contrary to our third hypothesis, our results showed that older age and time spent in the practice (a surrogate of experience) were inversely correlated with emotional exhaustion and depersonalization, and positively correlated with personal accomplishment among GPs and residents. Furthermore, regression analyses suggested a predictive role for age and time spent in practice (a surrogate of experience) in reducing emotional exhaustion and depersonalization among GPs, respectively. Our findings therefore support the line of evidence that stipulates a significant association between age as well as age-related experience and burnout. However, our findings should be interpreted with caution because of the potential issue of survival bias, i.e. those who burn out early in their careers are likely to quit their jobs, leaving behind the survivors who consequently exhibit lower levels of burnout. A meta-analysis conducted by Brewer and Shapard [51] showed a weak but significant negative correlation between employee age and emotional exhaustion. Based on these results and our findings, we suggest that age should be included in the risk profile for GPs.

In terms of our fourth hypothesis, we found evidence that a higher number of children (a surrogate of dependant care) was a significant correlate of burnout among female physicians. Our findings provide empirical support for the line of evidence, which suggests that dependant care (e.g., child rearing, looking after old parents) provided by working women is a source of chronic stress and ensuing burnout [11]. A plausible explanation for our findings could be that Hungarian female GPs may experience work-family conflict, a type of distress, which arises from the inability to balance the demands at work and at home. This could be due to the lack of efficient family-friendly policies in the Hungarian medical profession and the typically long working hours, which make combining work and family roles difficult for female physicians and particularly to physician mothers. Thus, female physicians in Hungary often find themselves compelled to fulfil a number of roles (mother, spouse, doctor) to the highest standards concurrently. This leads to work-family conflict and subsequently to burnout [35].

Our results confirm our fifth hypothesis, which stated that the number of patients in the practice (a surrogate of workload) positively correlated with of burnout. In particular, we identified this relationship for depersonalization among female physicians. There is a large body of evidence which shows that workload is a significant risk factor of burnout [12]. Work overload or high work demands, especially when occurs with lack of control and support, has been shown to predict emotional exhaustion, a central component of burnout [8]. On the other hand, lack of job resources has been theorized to account for depersonalization or disengagement [39] dimension of burnout. There is evidence to suggest that both of these processes occur at the same time. Based on our results, we posit that emotional exhaustion caused by work overload may augment disengagement and depersonalization, which is further corroborated by the lack of job resources among female GPs such as organizational policies to help balance work and home domain activities in a traditional society where female physicians are expected to fulfil multiple roles. This hypothesis would fit Leiter’s model of burnout, which states that emotional exhaustion leads to depersonalization and cynical attitudes toward work as employees attempt to remove themselves emotionally from their job as a way of coping with stress [46].

Our study has some limitations such as its cross-sectional nature, which includes biases of self-reporting and the inability to confer causality. In addition, our study sample might only be representative of GPs working in adult practices but not of GPs in paediatric and mixed practices. Also, whilst the response rate was high, non-responders might have biased the results as they might not suffer from burnout, hence burnout levels in the study might have been overestimated. Furthermore, we used arbitrary cut-off scores to classify general practitioners into cohorts reporting high, intermediate and low levels of burnout on the three dimensions. Whilst this method is useful to explore the objectives of the study in the research setting, it cannot be directly extrapolated into the clinical setting to support diagnosis of burnout.

Despite the limitations, our study has important strengths and significance for the practice. First of all, it is one of the first studies to explore psychosocial correlates and/or predictors of burnout among GPs in Hungary, plagued with severe shortage of physicians in almost every large specialty, and a rapidly ageing GP population. Furthermore, our study is one of the few investigations that explore the prevalence and potential predictors of burnout among residents [52]. In line with this line of research, our results confirm the multicausal nature of burnout among medical residents. In addition, our study provides a base for further explorations by virtue of generating hypotheses and developing research strategies for deploying effective interventions. In particular, recent research on the applicability of the MBI scales for supporting the diagnosis and management of burnout in the clinical setting based on a multi-dimensional model (a combination of the highest reported score on one of the three dimensions of burnout and various degrees of burnout reported on the remaining two scales) has suggested the development of five burnout profiles such as ‘engaged’, ‘ineffective’, ‘overextended’, ‘disengaged’, and ‘burnout’ [53]. Our results, in combination with our previous research, suggest that Hungarian GPs may fall into the ‘ineffective’ profile, which is characterised by low scores on the personal accomplishment scale as the leading burnout dimension. One of the objectives of these burnout profiles is to aid the development of appropriate interventions. Based on our results and those of others about the specific predictors of various burnout dimensions, we suggest that GPs with an ‘ineffective’ profile would benefit more from interventions that address resources, social support, and job satisfaction than from interventions that focus on reducing work overload or strengthening values, which have been associated with the ‘overextended’ and ‘disengaged’ burnout profiles, respectively.

## Conclusions

Our study provides insights into the potential gender-specific and psychosocial correlates of the different burnout dimensions among GPs and residents in Hungary and thus sets forth future directions not only for further gender, cross-cultural, and occupational research but also for specific interventions and preventive measures to reduce physician burnout and subsequent physician impairment and, thus, to improve retention and recruitment of GPs, and ultimately the quality of primary care.

## Abbreviations

CME: Continued Medical Education
GP: General Practitioner
MBI-HSS: Maslach Burnout Inventory
